# Short-channel field-effect transistors with 9-atom and 13-atom wide graphene nanoribbons

**DOI:** 10.1038/s41467-017-00734-x

**Published:** 2017-09-21

**Authors:** Juan Pablo Llinas, Andrew Fairbrother, Gabriela Borin Barin, Wu Shi, Kyunghoon Lee, Shuang Wu, Byung Yong Choi, Rohit Braganza, Jordan Lear, Nicholas Kau, Wonwoo Choi, Chen Chen, Zahra Pedramrazi, Tim Dumslaff, Akimitsu Narita, Xinliang Feng, Klaus Müllen, Felix Fischer, Alex Zettl, Pascal Ruffieux, Eli Yablonovitch, Michael Crommie, Roman Fasel, Jeffrey Bokor

**Affiliations:** 1 Department of Electrical Engineering and Computer Sciences, University of California, Berkeley, CA 94720 USA; 2 Materials Sciences Division, Lawrence Berkeley National Laboratory, Berkeley, CA 94720 USA; 3 Empa, Swiss Federal Laboratories for Materials Science and Technology, Überlandstrasse 129, Dübendorf, CH-8600 Switzerland; 4 Department of Physics, UC Berkeley, Berkeley, CA 94720 USA; 5 Flash PA Team, Semiconductor Memory Business, Samsung Electronics Co. Ltd., Gyeonggi-do, Korea; 6 Max Planck Institute for Polymer Research, Ackermannweg 10, Mainz, 55128 Germany; 7 Center for Advancing Electronics Dresden, Department of Chemistry and Food Chemistry, TU Dresden, Mommsenstrasse 4, Dresden, 01062 Germany; 8 Department of Chemistry, UC Berkeley, Berkeley, CA 94720 USA; 9 Kavli Energy NanoSciences Institute at the University of California, Berkeley and the Lawrence Berkeley National Laboratory, Berkeley, CA 94720 USA; 10 Department of Chemistry and Biochemistry, University of Bern, Freiestrasse 3, CH-3012 Bern, Switzerland

## Abstract

Bottom-up synthesized graphene nanoribbons and graphene nanoribbon heterostructures have promising electronic properties for high-performance field-effect transistors and ultra-low power devices such as tunneling field-effect transistors. However, the short length and wide band gap of these graphene nanoribbons have prevented the fabrication of devices with the desired performance and switching behavior. Here, by fabricating short channel (*L*_ch_ ~ 20 nm) devices with a thin, high-*κ* gate dielectric and a 9-atom wide (0.95 nm) armchair graphene nanoribbon as the channel material, we demonstrate field-effect transistors with high on-current (*I*_on_ > 1 μA at *V*_d_ = −1 V) and high *I*_on_ */I*_off_ ~ 10^5^ at room temperature. We find that the performance of these devices is limited by tunneling through the Schottky barrier at the contacts and we observe an increase in the transparency of the barrier by increasing the gate field near the contacts. Our results thus demonstrate successful fabrication of high-performance short-channel field-effect transistors with bottom-up synthesized armchair graphene nanoribbons.

## Abstract (web summary)

Graphene nanoribbons show promise for high-performance field-effect transistors, however they often suffer from short lengths and wide band gaps. Here, the authors use a bottom-up synthesis approach to fabricate 9- and 13-atom wide ribbons, enabling short-channel transistors with 10^5^ on-off current ratio.

## Introduction

The electronic, optical and magnetic properties of graphene nanoribbons (GNRs) can be engineered by varying their width and edge structure^1–13^. However, traditional methods to pattern GNRs, such as unzipping carbon nanotubes or lithographically defining GNRs from bulk graphene, yield GNRs with rough edges that degrade electronic transport^14^. Recent experiments have demonstrated bottom-up chemical synthesis of ultra-narrow ( < 2 nm) GNRs with uniform width and atomically-precise edges, in which the width and edge structure of the GNR is determined by the oligophenylene used in the polymerization step^1, 2, 4, 7, 9, 10^. This synthetic uniformity produces GNRs with high structural and electronic homogeneity, which is required for integration of GNR field-effect transistors (FETs) into large-scale digital circuits^15^. However, the wide band gap and short length of bottom-up synthesized GNRs have prevented the realization of high-performance FETs^16–19^.

Here, we show FETs with 9-atom and 13-atom wide armchair GNRs (9AGNRs and 13AGNRS, respectively). The band gap of ultra-narrow GNRs is very sensitive to its dielectric environment. For instance, the band gap of 7AGNRs obtained via scanning tunneling spectroscopy can vary by as much as 1 eV, depending on whether the substrate is conductive or insulating^8^. Since our GNR devices are on an insulating substrate, the band gap of the GNRs will be closest to the density functional theory band gap with GW correction^8, 12^. With a predicted band gap of 2.10 eV for the isolated 9AGNR and 2.35 eV for the 13AGNR^12^, these are the narrowest band gap GNRs that have been synthesized on a surface with useful length for device fabrication (5AGNRs have a smaller band gap but are only  ~ 10 nm long with current synthetic methods^6^). Thus, we were able to demonstrate high on-current (*I*_on_ > 1 μA at *V*_d_ = −1 V) and high *I*_on_ */I*_off_ ~ 10^5^ FETs at room temperature by using 9AGNRs as the channel material and a thin high-*κ* gate dielectric.

## Results

### Synthesis and transfer of GNRs

**Fig. 1:**
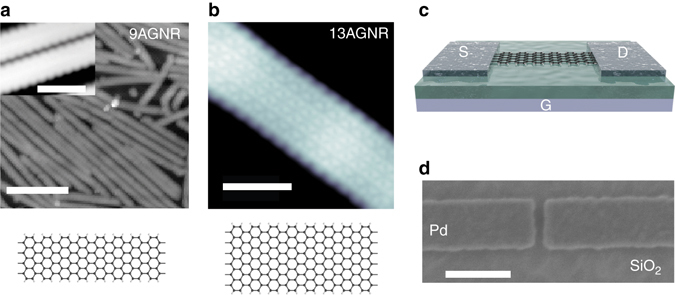
High-resolution STM GNR characterization and FET structure. **a** STM image of synthesized 9AGNR on Au with a *scale bar* of 10 nm (*V*_s_ = 1 V, *I*_t_ = 0.3 nA). *Inset*: High-resolution STM image of 9AGNR on Au (*V*_s_ = 1 V, *I*_t_ = 0.5 nA) with a *scale bar* of 1 nm. **b** High-resolution STM image of 13AGNR on Au with a *scale bar* of 2 nm (*V*_s_ = −0.7 V, *I*_t_ = 7 nA). **c** Schematic of the short channel GNRFET with a 9AGNR channel and Pd source-drain electrodes. **d** Scanning electron micrograph of the fabricated Pd source-drain electrodes with a *scale bar* of 100 nm

To synthesize the GNRs, the requisite monomer was evaporated onto a Au(111) surface under ultra-high vacuum and heated until it polymerized. Heating the substrate further causes individual polymers to planarize into GNRs (cyclodehydrogenation). The high quality of the GNRs is verified by high-resolution scanning tunneling microscope (STM)^1, 2^ imaging as shown in Fig. 1a, b.

**Fig. 2:**
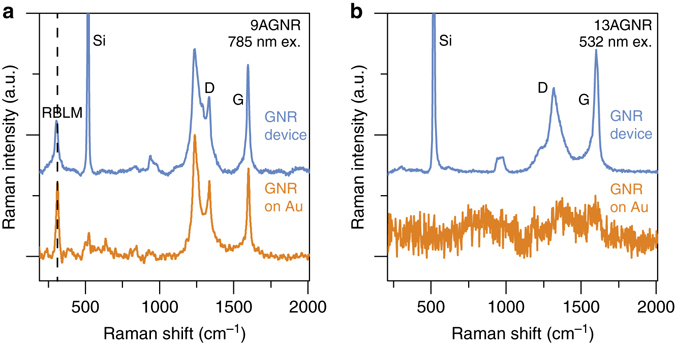
Raman spectra of as-grown GNRs on Au and GNRs after transfer and device processing. Raman spectra of **a** 9AGNRs and **b** 13AGNRs on the Au(111) growth substrate and after device fabrication shows that the GNRs remain intact. Since the excitation is off-resonance with the 13AGNR absorption, the Raman signal is weak on Au and the RBLM is not visible

**Fig. 3:**
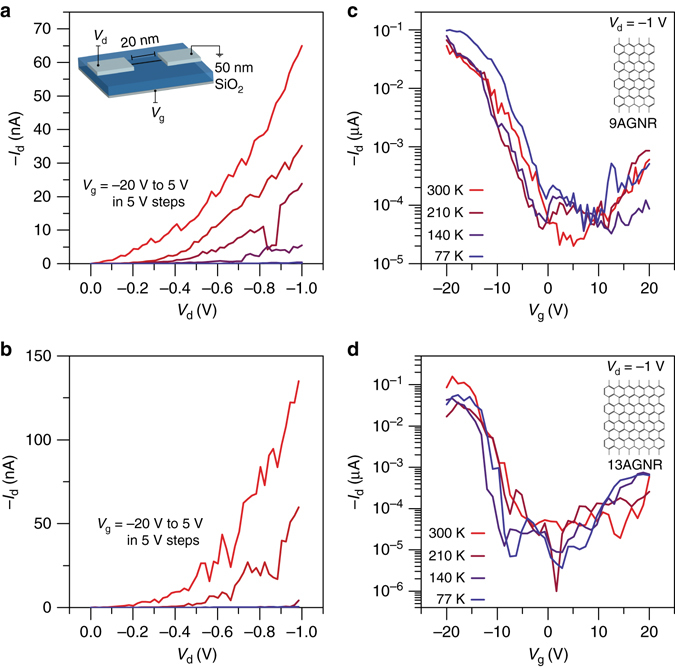
Transport characteristics of 9AGNRs and 13AGNRs gated with 50 nm SiO_2_ gate oxide. The presence of a SB is confirmed by non-linear current behavior at low drain bias and lack of current saturation at high drain bias for both **a** 9AGNRs and **b** 13AGNRs. The weak temperature dependence in the *I*_d_ *−V*_g_ behavior in **c** 9AGNRs and **d** 13AGNRs indicates that tunneling through the Pd-GNR SBs is the limiting transport mechanism of the device

Fabrication of GNRFETs requires the transfer of GNRs from the Au growth surface onto an insulating surface and subsequent device fabrication steps, as described in the Methods. Unfortunately, standard imaging techniques (atomic force microscopy, scanning electron microscopy, transmission electron microscopy, etc.) were not useful in imaging single GNRs on insulating surfaces due to the GNR’s small dimensions ( ~ 30 nm long,  ~ 1 nm wide and < 1 nm thick). Instead, we used Raman spectroscopy in order to verify that the structural integrity of the GNRs is maintained throughout the transfer and device fabrication process. As shown in Fig. 2a, the Raman spectrum with 785 nm wavelength excitation of the processed 9AGNRs looks identical to the spectrum taken of the as-grown 9AGNRs on Au. The presence of the radial breathing-like mode (RBLM) peak (311.5 cm^−1^) is evidence that the GNR width and edge structure is intact throughout device processing^20, 21^. Unlike 9AGNRs, the RBLM is not visible for the 13AGNR spectrum for either 532 or 785 nm excitation wavelengths due to off-resonance of the excitation (Fig. 2b and Supplementary Fig. 2). Still, the 13AGNRFETs were processed with the same fabrication steps as the 9AGNRFETs and both types of devices exhibit similar transport characteristics (Fig. 3).

### 13AGNRFETs and 9AGNRFETs with thick gate dielectrics

First, we fabricated devices with a nominal 20 nm channel length and a 50 nm SiO_2_ gate dielectric as illustrated in Fig. 3a. Using the same fabrication methods we made two different types of samples: one with 9AGNRs and one with 13AGNRs. After patterning  ~ 300 pairs of electrodes in the transferred GNR area, each defined channel was biased and tested for gate modulation of the current to find devices bridged by a GNR. Of the 300 devices, 28 devices and 29 devices were successfully fabricated for 9AGNR and 13AGNRs, respectively. Taking into account the length distribution of GNRs on the surface, this  ~ 10% ratio of bridged contacts to open devices indicates that almost all of the devices found contain one GNR in the channel as demonstrated by Supplementary Fig. 1 and Supplementary Note 1. Finally, GNR-GNR transport was ruled out by confirming that pairs of electrodes with gaps 30 nm or longer showed no conduction.

These 9AGNRFETs and 13AGNRFETs, as shown in Fig. 3 and Supplementary Fig. 3, showed similar electrical behavior due to their similar band gap. The presence of a Schottky barrier (SB) at the Pd-GNR interface is evident by the non-linear behavior at low bias in the *I*_d_ *–V*_d_ characteristics^22^, shown in Fig. 3a, b. To determine the contributions of thermionic vs. tunneling current across the SB, we measured the devices in vacuum at 77, 140, 210, and 300 K. As demonstrated in Fig. 3c, d, there is no significant change in the characteristics at these different temperatures for either 13AGNRFETs or 9AGNRFETs and the off-state current is at the gate leakage level (Supplementary Fig. 4). The weak temperature dependence in the current-voltage characteristics suggests that the limiting transport mechanism is tunneling through the barrier as opposed to thermionic emission over the barrier at the contacts^23, 24^. If thermionic emission was a significant transport mechanism across the SB, the current should be exponentially dependent on the inverse of temperature. Furthermore, the ambipolar behavior observed at low temperatures is only realistically possible with tunneling contacts, since thermally activated current is suppressed for electrons in a semiconductor with a band gap of > 2 eV. Tunneling contacts with weak temperature dependence have been observed for carbon nanotube FETs and other low-dimensional materials and verified via simulations^23–26^. Yet, the *I*_on_ ~ 100 nA in the devices shown in Fig. 3 is too low for high-performance applications, so the transmission through the SBs must be enhanced to improve the current.

### Ionic liquid gating of 9AGNRFETs

**Fig. 4:**
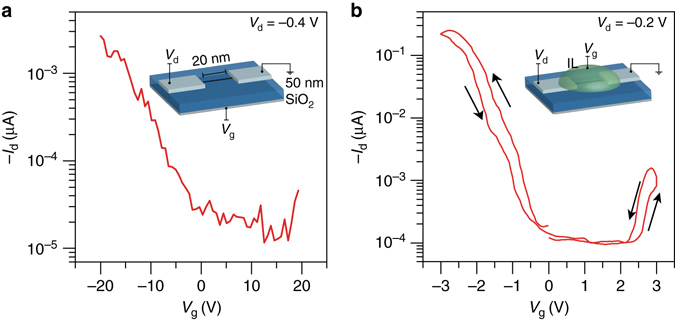
Ionic liquid gating of a 9AGNRFET at room temperature. **a** *I*_d_ *−V*_g_ characteristics of the device gated by the thick 50 nm SiO_2_ gate oxide. **b** *I*_d_ *−V*_g_ characteristics of the device gated with the ionic liquid which shows clear ambipolar behavior and improved on-state performance. *Inset*: ionic liquid (DEME-TFSI) gated 9AGNRFET device schematic

Ionic liquid (IL) gating has been previously used to improve the transparency of the SBs in MoS_2_^27^. Thus, we used the IL *N*,*N*-diethyl-*N*-(2-methoxyethyl)-*N*-methylammonium bis(trifluoromethylsulphonyl-imide) (DEME-TFSI) to improve the electrostatic coupling between the gate electrode and the GNR channel, increase the field at the Pd-GNR interface and improve the transmission through the barriers. The *I*_d_ *–V*_g_ behavior of a 9AGNRFET with IL gating is shown in Fig. 4b. This device shows clear enhancement in the on-current to  ~ 200 nA at −0.2 V drain bias (as opposed to 3 nA at −0.4 V for the 50 nm SiO_2_ dielectric device presented in Fig. 4a). The transistor also switches at smaller gate voltages due to the high gate efficiency of the IL.

### 9AGNRFETs with a thin gate dielectric

**Fig. 5:**
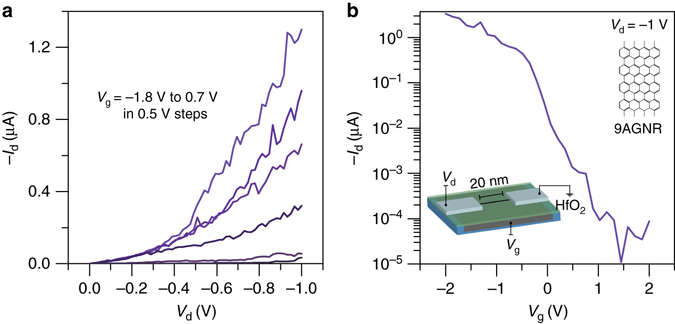
Transport characteristics of a scaled, high-performance 9AGNRFET at room temperature. **a** *I*_d_ *−V*_d_ characteristics of the scaled 9AGNRFET. **b** *I*_d_ *−V*_g_ of the devices show high *I*_on_ > 1 μA for a 0.95 nm wide 9AGNR and high *I*_on_ */I*_off_ ~ 10^5^. *Inset*: scaled 9AGNRFET schematic

Since solid dielectric gates must be used for logic device applications, we fabricated scaled 9AGNR devices with a thin HfO_2_ gate dielectric (effective oxide thickness of around 1.5 nm) as shown in Fig. 5. Resembling the IL device, the local HfO_2_ back gate is more efficient at improving transmission through the SB than the thick SiO_2_ global back gate^28^. As demonstrated by the *I*_d_ vs. *V*_g_ shown in Fig. 5, the device exhibits excellent switching characteristics, *I*_on_/*I*_off_ ~ 10^5^, and a high *I*_on_ ~ 1 μA at *V*_d_ = −1 V. The SB is still prominent as indicated by the non-linear *I*_d_ vs. *V*_d_ characteristics at low bias and the large subthreshold swing (SS) in this device ( ~ 350 mV/dec). Even if tunneling is enhanced by using the thin gate oxide, large SB devices tend to suffer from SS and *I*_on_ degradation^22, 24^.

Assuming most of the current is transported by an individual GNR in the channel, the local back gated device performance corresponds to a 0.95 nm GNR-width (100 nm electrode width) normalized current drive of  ~ 1000 μA/µm ( ~ 10 μA/µm) at −1 V drain bias. Although some top-down GNRs have higher normalized conductance, the GNRs used here have much larger band gaps^29–32^. Therefore, we were able to scale our devices to short channel lengths without affecting the *I*_on_/*I*_off_. At the same time, the impact of the large SBs on *I*_on_ was minimized with the improved gate efficiency of the thin high-k dielectric. The expected value of > 20 mA/µm in GNR devices with no SB^33^ could be attainable by integrating bottom-up synthesized GNRs with smaller band gaps ( ~ 1 eV) and densely aligned GNRs^34^.

## Discussion

We thus successfully demonstrate short-channel FETs with bottom-up synthesized armchair GNRs with excellent switching behavior and on-state performance by aggressively scaling the gate dielectric. Bottom-up GNR devices are, therefore, good candidates for high-performance logic applications, especially with advances in densely aligned GNR synthesis^34^ as well as narrow band gap GNR growth^6^. These results motivate the study of GNR growth mechanism in order to improve the average length of GNRs and increase device yield. Our methodology can be applied to other exotic device structures as well, such as tunnel FETs, which incorporate atomically precise GNR heterostructures^9, 10, 35^.

## Methods

### 9AGNR growth

9AGNRs were synthesized from 3′,6′-dibromo-1,1′:2′,1″-terphenyl precursor monomers^7^. First, the Au(111)/mica substrate (200 nm Au; PHASIS, Geneva, Switzerland) was cleaned in ultra-high vacuum by two sputtering/annealing cycles: 1 kV Ar^+^ for ten minutes followed by a 470 °C anneal for ten minutes. Next, the monomer was sublimed onto the Au(111) surface at a temperature of 60–70 °C, with the substrate held at 180 °C. After 2 min of deposition (resulting in approximately half monolayer coverage), the substrate temperature was increased to 200 °C for ten minutes to induce polymerization, followed by annealing at 410 °C for ten minutes in order to cyclodehydrogenate the polymers and form 9-AGNRs.

### 13AGNR growth

13AGNRs were synthesized using 2,2′-Di((1,1′-biphenyls)-2-yl)-10,10′-dibromo-9,9′-bianthracene building blocks^2^. Similar to the 9AGNR substrate, the Au(111)/mica substrate (200 nm Au; PHASIS, Geneva, Switzerland) is cleaned in ultra-high vacuum by two sputtering/annealing cycles: 1 kV Ar^+^ for ten minutes followed by a 450 °C anneal for ten minutes. The monomer was sublimed at 222 °C onto the clean substrate held at room temperature. The sample was then slowly annealed stepwise to 340 °C to form 13AGNRs.

### Preparation of 50 nm SiO_2_ back gates

Using dry oxidation, 50 nm SiO_2_ was grown on heavily doped 150 mm silicon wafers. Alignment markers and large pads for electrical probing were patterned using standard photolithography and lift-off patterning of 3 nm Cr and 25 nm Pt. The wafer was then diced and individual chips were used for GNR transfer and further device processing.

### Preparation of 6.5 nm HfO_2_ local back gates

Using dry oxidation, 100 nm SiO_2_ was grown on 150 mm silicon wafers. The local back gates were lithographically patterned and dry etched into the SiO_2_ followed by lift-off of 3 nm Ti and 17 nm Pt^36^. 6.5 nm HfO_2_ was grown in an atomic layer deposition system at 135 °C. Alignment markers and large pads for electrical probing were patterned using standard photolithography and lift-off of 3 nm Cr and 25 nm Pt. The wafer was then diced and individual chips were used for GNR transfer and further device processing.

### GNR transfer and patterning of source-drain electrodes

GNR/Au/mica was floated in 38% HCl in water, which caused the mica to delaminate with the Au film remaining floating on the surface of the acid^10^. The floating gold film was picked up with the target substrate, with the GNRs facing the dielectric surface. Subsequent gold etching in KI/I_2_ yielded isolated, randomly distributed GNRs with sub-monolayer coverage on the target substrate. After the GNR transfer, poly-methyl methacrylate (molecular weight 950 K) was spun on the chips at 4 Krpm and followed by a 10 min bake at 180 °C. ~ 300 source drain electrodes (100 nm wide, 20 nm gaps) were patterned using a JEOL 6300-FS 100 kV e-beam lithography system and subsequently developed in 3:1 IPA:MIBK at 5 °C. In all, 10 nm Pd was deposited using e-beam evaporation and lift-off was completed in Remover PG at 80 °C.

### Raman characterization

Raman characterization of the 9AGNR was performed with a Bruker SENTERRA Raman microscope using a 785 nm diode laser with 10 mW power and a 50× objective lens, resulting in a 1–2 micrometer spot size. No thermal effects were observed under these measurement conditions and an average of three spectra from different points was made for each sample. Raman measurements of the 13AGNR were made with a Horiba Jobin Yvon LabRAM ARAMIS Raman microscope using 532 and 785 nm diode lasers with 10 mW power each and a 50× objective lens, resulting in a 1–2 micrometer spot size. No thermal effects were observed under these measurement conditions and an average of five spectra from different points was made for each sample.

### Electrical characterization

Devices were first screened in ambient conditions using a cascade probe station and an Agilent B1500A parameter analyzer. Vacuum and variable temperature measurements were then performed in a Lakeshore probe station. IL devices were measured with a *V*_g_ sweep speed of 50 mV/s.

### Data availability

The data that support the findings of this study are available from the corresponding author upon reasonable request.

## Electronic supplementary material

Supplementary Information
