# Supplementary material for: Short-channel field-effect transistors with 9-atom and 13-atom wide graphene nanoribbons
Source: Nat Commun. 2017 Sep 21;8:633. doi: 10.1038/s41467-017-00734-x (PMC5608806; doi:10.1038/s41467-017-00734-x)
Supplement: Supplementary file 1 — Supplementary Information [file 41467_2017_734_MOESM1_ESM.pdf]

Supplementary Note 1: extraction of number of GNRs in the channel

We used a Monte Carlo simulation to estimate the number of GNRs in our device channels based on our device yield. Assuming a uniform spatial distribution of GNRs, we simulate the expected device yield and distribution of number of GNRs in the channel. The input parameters of the simulation were the GNR number density on the surface and GNR length. We varied these parameters to generate Fig. S1. With the experimentally obtained yield of ~10%, the percentage of devices with more than 1 GNR in the channel goes as high as 8% for higher surface density and 4% for low surface density. Out of the devices with multiple GNRs, an insignificant percentage has more than 2 GNRs/channel. Thus, we estimate that only 1-3 devices out of ~30 fabricated devices have 2 GNRs in the channel. However, due to the tendency for GNRs to be locally aligned to each other, as shown in the STM image, we are underestimating the number of GNRs in the channel. We believe these devices would not account of the high tail end of the on-current distribution, since both GNRs would have to have good contact length under the Pd contacts to improve conduction over a single GNR channel with a large contact length.

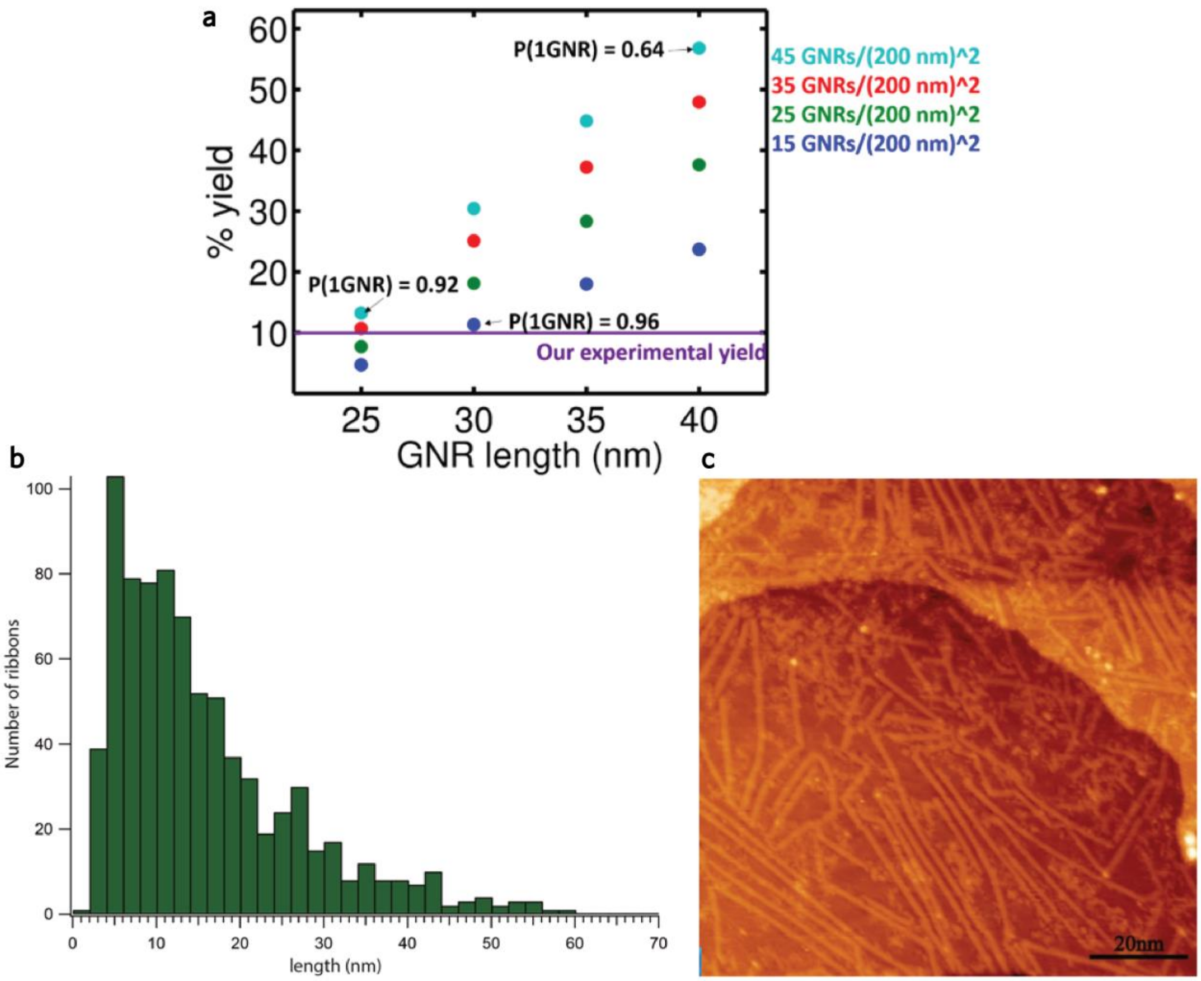

Supplementary Figure 1. (a) Simulated % yield of working devices as a function of GNR length and number density. The P(1GNR) values denote the probability of a yielded device to have a single GNR in the channel. With our ~10% experimental yield, we estimate that only 1-3 devices out of 30 contain 2 GNRs in the channel. (b) Length histogram of GNRs shows that only 11% of GNRs in our typical growths have a length of >30 nm. (c) Representative STM images from the series of images used to obtain the 9AGNR length histogram.

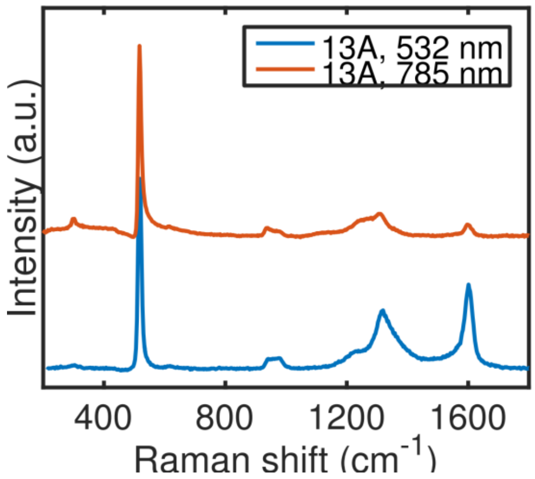

Supplementary Figure 2. Raman spectra of 13AGNRs after device fabrication using 532 nm and 785 nm wavelength excitation. The RBLM is not detectable under these excitation conditions due to excitation off-resonance effects.

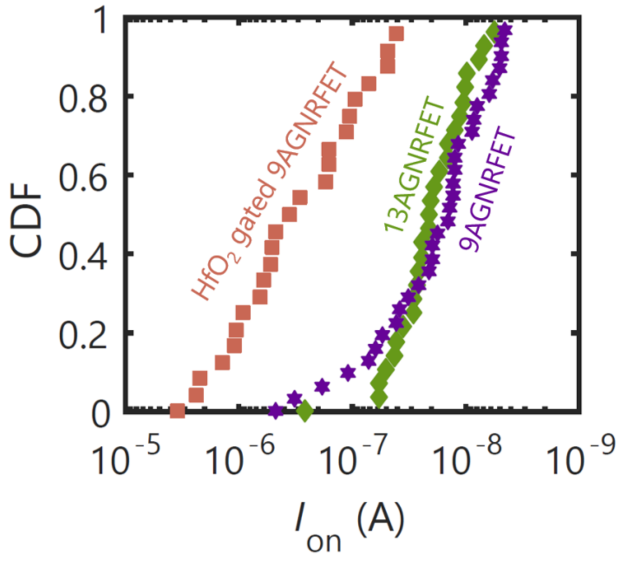

Supplementary Figure 3. Cumulative distribution function (CDF) of I_{on} in 13AGNRFETs and 9AGNRFETs with 50 nm SiO_2 gate dielectrics and 9AGNRFETs with thin HfO_2 gate dielectrics. The CDF is defined as the total fraction of devices with on-current greater than the given value of I_{on}. Both types of devices have similar behavior due to the similar band gap and variations in on-state performance are most likely due to variations in the overlap length between the Pd and GNR and variations in the channel length.

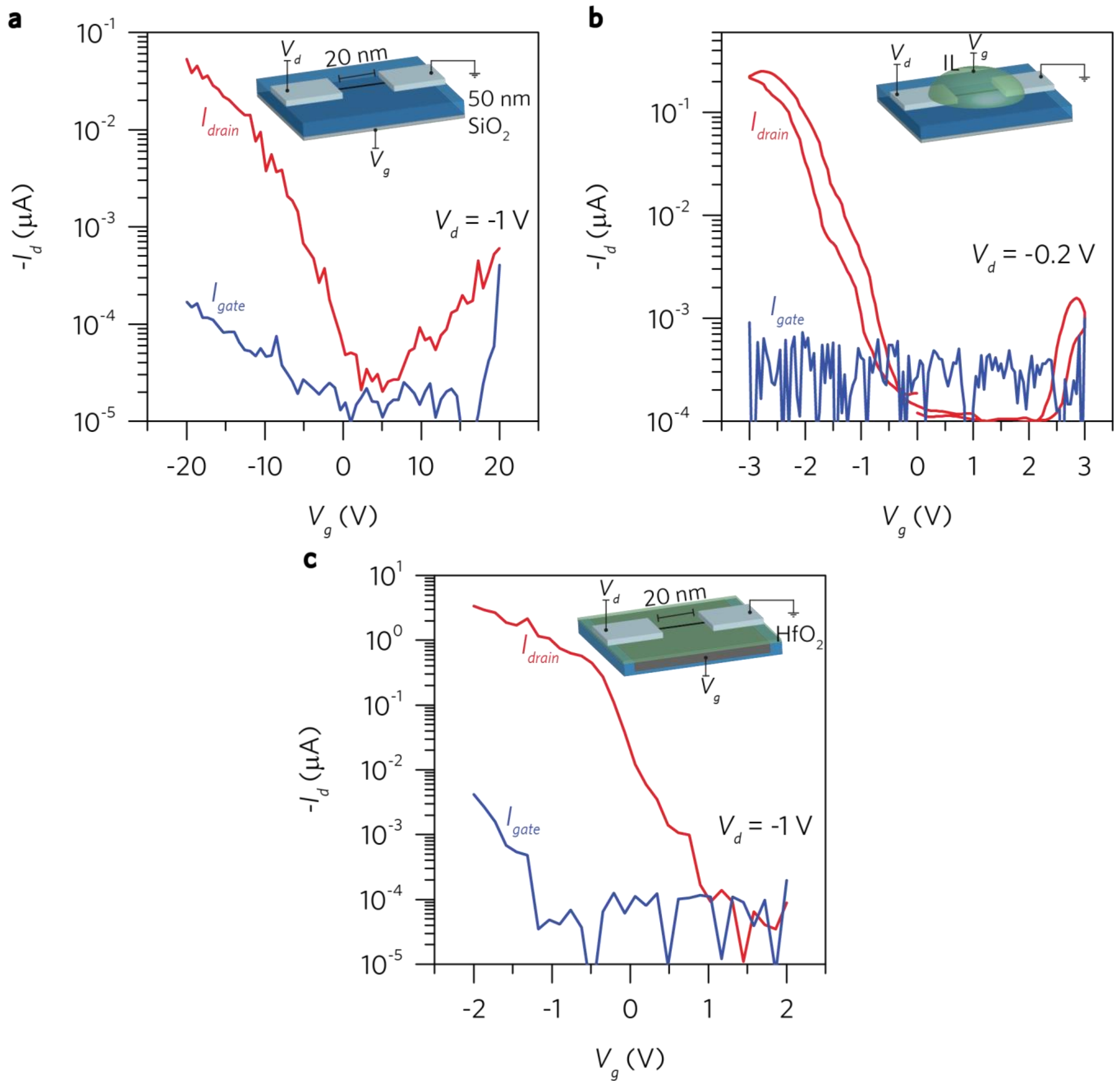

Supplementary Figure 4. (a) I_d-V_{bg} characteristics of the 9AGNRFET shown in Fig 3 in the main text which shows that the gate leakage limits the off-current. (b) I_d-V_{bg} characteristics of the IL gated 9AGNRFET, which shows a gate leakage of <1 nA which is typical for this type of gating technique[1]. (c) I_d-V_{bg} characteristics of the 9AGNRFET with a thin HfO_2 gate dielectric that shows the off-current is limited by gate leakage.

Supplementary References

1. Ye, J. T. *et al.* Superconducting Dome in a Gate-Tuned Band Insulator. *Science* **338,** 1193–1196 (2012).
